# Regularized regression method for genome-wide association studies

**DOI:** 10.1186/1753-6561-5-S9-S67

**Published:** 2011-11-29

**Authors:** Jin Liu, Kai Wang, Shuangge Ma, Jian Huang

**Affiliations:** 1 Department of Statistics and Actuarial Science, University of Iowa, 241 Schaeffer Hall, Iowa City, IA 52242, USA; 2 Department of Biostatistics, University of Iowa, C22 General Hospital, Iowa City, IA 52242, USA; 3 Division of Biostatistics, School of Public Health, Yale University, 60 College Street, New Haven, CT 06520, USA

## Abstract

We use a novel penalized approach for genome-wide association study that accounts for the linkage disequilibrium between adjacent markers. This method uses a penalty on the difference of the genetic effect at adjacent single-nucleotide polymorphisms and combines it with the minimax concave penalty, which has been shown to be superior to the least absolute shrinkage and selection operator (LASSO) in terms of estimator bias and selection consistency. Our method is implemented using a coordinate descent algorithm. The value of the tuning parameters is determined by extended Bayesian information criteria. The leave-one-out method is used to compute *p*-values of selected single-nucleotide polymorphisms. Its applicability to a simulated data from Genetic Analysis Workshop 17 replication one is illustrated. Our method selects three SNPs (C13S522, C13S523, and C13S524), whereas the LASSO method selects two SNPs (C13S522 and C13S523).

## Background

Genome-wide association studies (GWAS) are a modern approach to genetic studies. Although GWAS successfully dissect genetic factors that underlie complex traits, they raise many challenging statistical issues. A prominent issue is how to identify single-nucleotide polymorphisms (SNPs) that are in linkage disequilibrium (LD) with a genetic variant of weak effect. To identify such SNPs, investigators use the modern approach of regularized regression, for instance, the least absolute shrinkage and selection operator (LASSO) [1]. However, existing regularized regression methods do not take into account LD information among adjacent SNPs. The fused LASSO [2] may be suitable for this purpose. However, the ambiguity in the choice of the reference allele for scoring genotypes makes it not applicable. Presumably, incorporating LD information into the analysis would be highly beneficial in delineating association signals by achieving smoothness and reducing randomness in single-SNP analysis. To make use of LD information, we have developed an L2 penalty that encourages a smaller difference in genetic effect at adjacent SNPs that are in stronger LD. This penalty is used in combination with the minimax concave penalty (MCP) [3], which is efficient in shrinking many nuisance predictors to exactly zero. In what follows, we describe the new method and then present its application to the Genetic Analysis Workshop 17 (GAW17) simulated data set of unrelated individuals.

## Methods

Let *p* be the number of SNPs and *n_j_* the number of subjects whose genotypes are nonmissing at the *j*th SNP. The centered phenotype of the *i*th subject with nonmissing genotype at SNP *j* is denoted *y_ij_*. The genotype at a SNP is scored as 0, 1, or 2 depending on the number of copies of the reference allele in the subject. Let *x_ij_* denote the standardized genotype scores satisfying Σ*_i_x_ij_* = 0. Then:(1)

Let *β_j_* be the genetic effect corresponding to SNP *j*. The model solves:(2)

There are two parts of penalty here, denoted *ρ*_1_ and *ρ*_2_. The first part is the MCP [3]*ρ*_1_(·; *λ*, *γ*), defined by:(3)

The MCP contains a soft threshold (*γ* = ∞) and a hard threshold (*γ* = 1) as special cases. *λ*_1_ is a tuning parameter. The second part of the penalty is the quadratic absolute difference in genetic effect between two successive SNPs:(4)

We choose *ς_j_* in expression (2) to be the absolute value of the Pearson correlation between the genotype scores of SNP *j* and SNP (*j* + 1). The second penalty was motivated by the fact that the adjacent SNPs are usually highly correlated.

**Figure 1:**
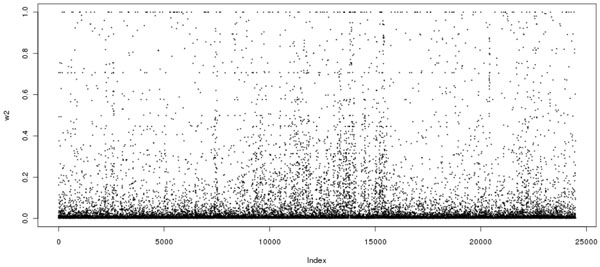
Absolute lag-one autocorrelation of SNPs over the genome

**Figure 2:**
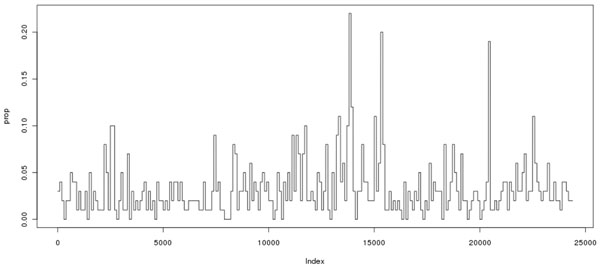
Proportion of absolute lag-one autocorrelation coefficients greater than 0.5 for 100 SNPs per segment over the genome

Figure 1 shows the absolute lag-one autocorrelation coefficients over the whole genome. Figure 2 shows the proportion of the absolute lag-one autocorrelation coefficients greater than 0.5 for 100 SNPs per segment over the genome. One can see that even for partially selected SNPs over the genome, strong correlations exist between adjacent SNPs. Although it may be more informative to use pairwise correlations among SNPs, the computational burden makes this implementation impossible in a real data set. Those facts motivated us to include the adjacent LD information in the second penalty in the model. The method is referred to as the smoothed minimax concave penalization (SMCP) [4].

The loss function in expression (2) is a sum of the marginal loss function at each SNP. We use a marginal loss function instead of a joint loss function because it is easier to deal with missing genotypes that way. Huang et al. [5] discussed the asymptotic properties of a marginal loss function with a bridge penalty under certain regularity conditions.

We implement an iterative coordinate descent algorithm to estimate model parameters. This algorithm has been used on many other occasions, including estimation in nonconvex penalized regression [6]. Because the first derivative of the objective function has explicit solutions, this algorithm is computational efficient. For the tuning parameters *λ*_1_ and *λ*_2_, we reparameterize them through:(5)(6)

The value of tuning parameter *γ* in the MCP is chosen to be 3 [6]. *η* is fixed at 0.1, and *τ* is determined by using the extended Bayesian information criterion (EBIC) [7]. We use the leave-one-out (LOO) method [8] to evaluate the significance of the selected SNPs.

## Results

The GAW17 data set consists of 24,487 SNP markers throughout the genome for 697 individuals. We analyze the unrelated individuals data with quantitative trait Q1 in replicate 1. All SNPs are included in the analysis. We coded the seven population groups as dummy variables. We first regress the quantitative trait Q1 on sex, age, smoking status, and group dummy variables in order to remove their confounding effects. This procedure helps to adjust for population stratification. Then, we use the residuals from this regression as the response and fit them using the SMCP model and the LASSO model. The selected tuning parameter *τ* is 1.655 for the SMCP model with *η* = 0.1 and 0.184 for the LASSO model.

**Figure 3:**
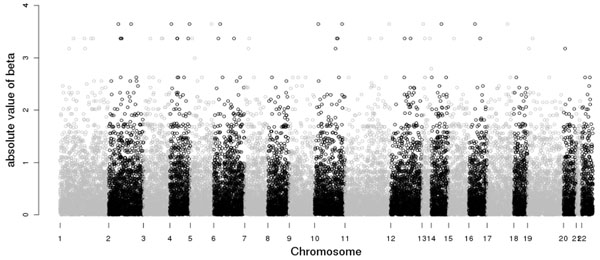
|*β*| estimates from single-SNP linear regression over the genome

**Figure 4:**
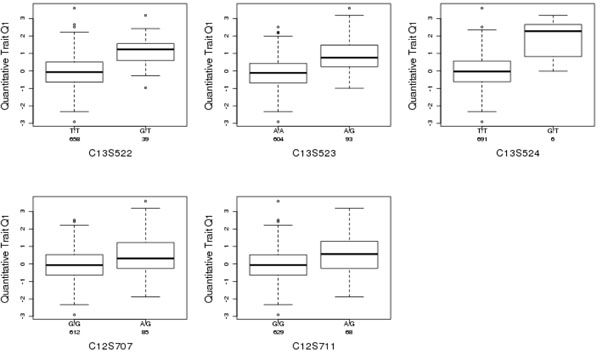
Boxplots for SNPs selected using the SMCP method

Absolute values of the estimates from the simple linear regression are plotted in Figure 3. The estimation results are presented in Table 1. Both the SMCP model and the LASSO model selected two SNPs (C13S522 and C13S523) from gene *FLT1*. For each method, these two SNPs have significant LOO *p*-values. The SMCP model selected three more SNPs, one (C13S524) from gene *FLT1* and the other two (C12S707 and C12S711) from gene *PRR4*. Only one SNP (C13S524) from gene *FLT1* is significant. The boxplots for these five SNPs selected by the SMCP and LASSO models are shown in Figure 4.

**Table 1:** SNPs selected by the SMCP and LASSO models for trait Q1 in replicate 1

SNP	Position	Gene	Univariate estimate	Univariate *p*-value	SMCP estimate	LOO *p*-value	LASSO estimate	LOO *p*-value
C12S707	11065657	*PRR4*	0.53	2.0 × 10^−7^	0.002	7.9 × 10^−1^		
C12S711	11065733	*PRR4*	0.61	7.6 × 10^−8^	0.007	3.0 × 10^−1^		
C13S522	27899910	*FLT1*	1.22	2.1 × 10^−17^	0.169	5.7 × 10^−7^	0.096	1.6 × 10^−8^
C13S523	27899912	*FLT1*	0.94	2.6 × 10^−22^	0.173	6.2 × 10^−10^	0.134	1.6 × 10^−13^
C13S524	27899915	*FLT1*	1.88	2.2 × 10^−7^	0.058	1.1 × 10^−2^		

With knowledge of the underlying model, we computed the true-positive rate and the false-positive rate for the SMCP model , the LASSO model, and regular single-SNP regression on trait Q1 using all 200 replicates (Table 2). For regular single-SNP regression, the Benjamini-Hochberg method is used to control the false discovery rate and to conduct multiple testing. The SMCP model tends to select more SNPs than the LASSO model with a higher true-positive rate and a higher false-positive rate. Although regular methods can select a higher true positive, its false positive is much higher than those in the SMCP and LASSO models. Further simulation studies can be found in [4].

**Table 2:** Mean and standard error (in parentheses) of true positives and false positives for selected SNPs over 200 replicates for trait Q1

	SMCP model	LASSO model	Regular regression
True positive	3.35 (1.52)	2.48 (1.19)	7.03 (1.81)
False positive	18.42 (36.20)	8.64 (21.53)	174.35 (87.87)

## Discussion

The penalized approach is a modern variable selection method developed to handle large *p*, small *n* problems. Application of this approach to GWAS is highly anticipated. Compared to traditional GWAS, in which SNPs are analyzed one by one, a penalized method is able to handle a collection of SNPs simultaneously. We have used a method that takes into account the LD information among adjacent SNPs in order to reduce the randomness seen in the traditional one-SNP-at-a-time analysis. For trait Q1 in replicate 1, the SMCP model selected three SNPs (C13S522, C13S523, and C13S524) from the associated gene *FLT1* and two SNPs that are false positives. In comparison, the LASSO model selected two SNPs (C13S522 and C13S523), both of which are true positives. We note that the SNPs provided for GAW17 are a small subset of the SNPs that are genotyped. The strength of LD for this set of SNPs has been greatly reduced. In addition, the GAW17 data were simulated to mimic rare variants. The SMCP method is specially designed to map rare variants. Even so, the SMCP model is able to select three SNPs, more than the LASSO model can. In comparison, the results of the regular simple linear regression are much noisier.

## Conclusions

The SMCP model is a novel penalized regression method. By taking into account the LD information between adjacent SNPs, the SMCP model is a useful tool that is better at delineating an association signal while reducing random noise. The algorithm used for the SMCP model is available in R package SMCP.

## Competing interests

The authors declare that there are no competing interests.

## Authors’ contributions

JL, JH and SM conceived of study. JL participated in the design and carried out the analysis and helped to draft the manuscript. KW helped to draft the manuscript. All authors read and approved the final manuscript.
